# Retroviral Restriction Factors and Their Viral Targets: Restriction Strategies and Evolutionary Adaptations

**DOI:** 10.3390/microorganisms8121965

**Published:** 2020-12-11

**Authors:** Guney Boso, Christine A. Kozak

**Affiliations:** Laboratory of Molecular Microbiology, National Institute of Allergy and Infectious Diseases, Bethesda, MD 20892-0460, USA; guney.boso@nih.gov

**Keywords:** retroviruses, restriction factors, viral evolution, viral antagonists

## Abstract

The evolutionary conflict between retroviruses and their vertebrate hosts over millions of years has led to the emergence of cellular innate immune proteins termed restriction factors as well as their viral antagonists. Evidence accumulated in the last two decades has substantially increased our understanding of the elaborate mechanisms utilized by these restriction factors to inhibit retroviral replication, mechanisms that either directly block viral proteins or interfere with the cellular pathways hijacked by the viruses. Analyses of these complex interactions describe patterns of accelerated evolution for these restriction factors as well as the acquisition and evolution of their virus-encoded antagonists. Evidence is also mounting that many restriction factors identified for their inhibition of specific retroviruses have broader antiviral activity against additional retroviruses as well as against other viruses, and that exposure to these multiple virus challenges has shaped their adaptive evolution. In this review, we provide an overview of the restriction factors that interfere with different steps of the retroviral life cycle, describing their mechanisms of action, adaptive evolution, viral targets and the viral antagonists that evolved to counter these factors.

## 1. Introduction

Retroviruses replicate by converting their single-stranded RNA genome into double-stranded DNA through a virus-encoded reverse transcriptase. These DNA copies are integrated into host chromosomes where they persist as a permanent part of the host cell genome. When retroviruses infect germline cells, integrated copies can be passed on to the next generation. These integrated proviruses are termed endogenous retroviruses (ERVs). As the retroviral genome encodes only a limited number of genes, replication of exogenous viruses and expression of ERVs rely on the host cell machinery. These viruses can be mutagenic and pathogenic, and this puts strong selective evolutionary pressure on the host genes appropriated for virus replication. The result has been the emergence of defensive genes that can inhibit viral infection and that have been derived from cellular genes, or more rarely, from ERVs. These restriction factors comprise the innate immune system that represents the first line of defense against viral attack.

While each restriction factor has followed a unique evolutionary path, some common themes have emerged (Table 1). First, most restriction factors are encoded by interferon-stimulated genes (ISGs) that are upregulated upon cellular exposure to type I or type II interferons (IFNs). Second, phylogenetic studies in the last two decades demonstrated that most restriction factors show signatures of positive or diversifying selection—that is, the rate of nonsynonymous mutations that result in amino acid substitutions is higher than the rate of synonymous mutations. The rapid evolution observed in the genes encoding retroviral restriction factors has often been countered by rapidly evolving viral evasive mechanisms which include virus-encoded antagonists and signatures of positive selection in the viral target of host restriction factors. This evolutionary conflict represents a genetic arms race described by the Red Queen hypothesis [1,2].

**Figure 1:**
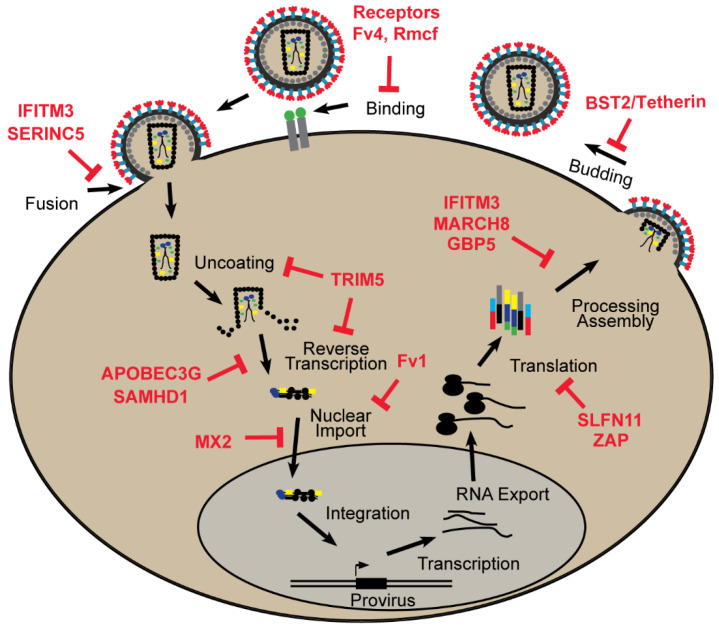
Restriction factors block specific stages of the retroviral life cycle.

Here, we describe a well-characterized set of retroviral restriction factors for which there is substantial information on their restriction mechanisms as well as their evolutionary history. The majority of these factors were discovered in primates or rodents through studies on HIV-1 which causes the acquired immune deficiency syndrome (AIDS) in humans, and on mouse leukemia viruses (MLVs) which induce lymphomas, immunodeficiencies, and neurological diseases. Some of these factors are restricted to specific taxonomic lineages, while others are carried by many mammalian species, showing broad antiviral activity against multiple retroviruses and in some cases against other families of viruses (Table 1). Decades of studies have produced an increased understanding of how these factors work and what stage of the replication cycle is blocked (Figure 1), and, in many cases, also describing the coevolutionary adaptations in the interacting host and virus proteins.

## 2. Retroviral Restriction Factors

### 2.1. Binding and Entry

Retroviruses enter susceptible cells through their interactions with specific cell surface receptors. These interactions can be inhibited by receptor polymorphisms as well as by other transmembrane proteins that interfere with binding or membrane fusion.

#### 2.1.1. Receptors

**Figure 2:**
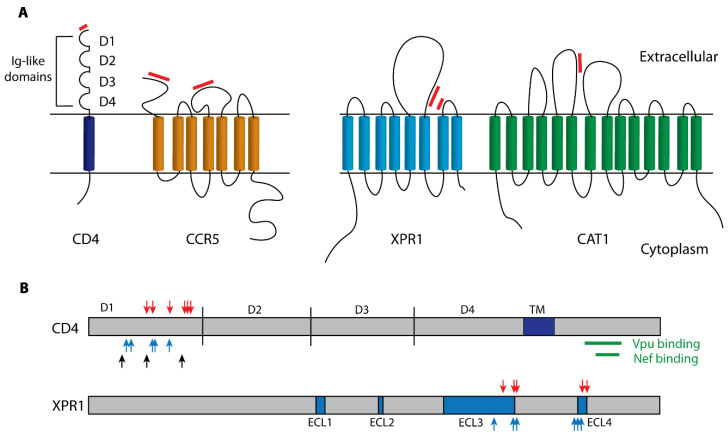
Structure and functional features of the HIV-1 and MLV cell surface receptors. (**A**) Schematic diagrams of the receptors for HIV-1 (CD4 and CCR5) and for different MLV subtypes (XPR1 and CAT1). Red bars indicate regions that bind virus envelope [7,16,17]. (**B**) XPR1 and CD4 receptor proteins. Blocks identify the transmembrane domain of CD4 and the extracellular loops (ECLs) in XPR1. Positively selected residues are marked with red arrows [13,17,18,19]. Receptor critical sites are marked with blue arrows and black arrows identify polymorphic sites in chimpanzee CD4 that influence SIV binding. Green bars identify CD4 sites susceptible to downregulation by Nef and Vpu [8,9,11,12].

Receptor-mediated restriction of virus entry has been documented for HIV-1 and MLVs. The HIV-1 envelope (Env) interacts with two membrane proteins for entry, a receptor, CD4, and one of two co-receptors, the chemokine receptors CCR5 and CXCR4 (Figure 2A) [3,4,5]. CD4 is a type I transmembrane protein that functions as a co-receptor for the T cell receptor signaling complex in response to antigen presentation by the MHC Class II molecules [6]. These proteins mediate HIV-1 infection of immune system cells, and receptor-functional orthologs are largely restricted to humans and related primates. CD4 binds virus through the D1 Ig domain, and CCR5 has two virus- binding domains in its N-terminus and in its second extracellular loop (Figure 2A) [7]. CD4 is downregulated in infected cells by the HIV-1 proteins Nef and Vpu (Figure 2B), and this prevents superinfection, thus avoiding apoptosis and production of infection-compromised virions, and also reduces sensitivity to inhibition by another restriction factor, SERINC5 [8,9,10]. While there are no known variants of *CD4* in humans that affect the efficiency of HIV-1 infection, *CD4* is highly variable in chimpanzees, and this variation is responsible for restricted susceptibility to SIV, the progenitor of HIV-1 (Figure 2B) [11,12]. Moreover, *CD4* has been molded by positive selection in primates with rapidly evolving residues found in the HIV-1 Env interacting interface of the CD4 protein, but not affecting the sites targeted by Vpu and Nef (Figure 2B) [13,14]. These findings suggest that co-evolution with SIVs has accelerated the evolution of *CD4* in primates. In some human populations, HIV-1 entry can be blocked by a variant of the *CCR5* coreceptor with a 32-base pair (bp) deletion in the second extracellular loop of the protein leading to the introduction of a premature stop codon which renders it non-functional as a co-receptor [15].

MLVs isolated from laboratory mice have host range subgroups that rely on two receptors, CAT1 for the ecotropic or mouse-tropic MLVs, and XPR1 for MLVs that can also infect other mammalian species (Figure 2A) [20,21]. These host genes function, respectively, as an amino acid transporter and a phosphate exporter [22,23,24]. CAT1 orthologs are functional as receptors only in mice, but wild mice have only recently been exposed to ecotropic MLVs, as these ERVs are found only in Eurasian and some California mice [25]. Only one mouse CAT1 sequence variant has been identified; the *Mus dunni* CAT1 restricts infection by Moloney MLV [26]. In contrast, the older XPR1-dependent MLV ERVs are found in all house mouse subspecies [25], and this extended exposure to virus challenge was accompanied by the evolution of six functional XPR1 variants in *Mus,* five of which restrict different subsets of MLVs. These restrictions result from deletions or substitutions in the two receptor determining regions of XPR1 (Figure 2), all of which were acquired by MLV-infected wild mouse populations (reviewed in [17]). This suggests that the mutant XPR1 variants have a survival advantage which is supported by an observed pattern of positive selection (Figure 2B) and also explains the co-evolution of viral Env variants with different receptor usage patterns [19]. Nonpermissive *Xpr1* orthologs are rare among mammals and birds but are found in a few mammalian species like hamsters [27], and in chickens, which were domesticated in India where their exposure to MLV-infected mice likely selected for inactivating XPR1 mutations [18]. A third MLV receptor, the Pit2 phosphate transporter [28,29], has no known functional polymorphisms in mice and is used by wild mouse amphotropic MLVs [30,31], a virus subtype that has not endogenized, and is found as infectious virus only in isolated mouse subpopulations in California [32].

Retrovirus entry can also be blocked by factors that interfere with receptor function (reviewed in [16,33]). The mouse genome contains several such resistance genes including *Fv4*, which blocks ecotropic MLVs, and *Rmcf* and *Rmcf2* which restrict XPR1-dependent MLVs (Figure 1). These genes have all been identified as ERVs that are defective but have intact *env* genes capable of producing trimeric proteins comprised of extracellular surface (SU) subunits that bind virus and the transmembrane (TM) subunit responsible for fusing host and viral membranes. *Fv4*, *Rcmf*, and *Rcmf2* are thought to mask or downregulate the activity of their cognate receptors, and *Fv4* additionally has a defect in the fusion peptide of the transmembrane domain of *env*, so virions that incorporate this Env have reduced infectivity [34]. This use of co-opted Env genes to block exogenous infection has also been described in chickens, sheep, and cats (reviewed in [33]).

#### 2.1.2. SERINC5

SERINC5 belongs to the serine incorporator (SERINC) gene family, a highly conserved group of genes found in all eukaryotes that encode 9-11 pass transmembrane proteins. SERINC proteins incorporate the amino acid serine into the lipids of cell membranes [35]. Mammals carry five SERINC genes while some lower eukaryotes, such as *C. elegans* and *S. cerevisiae* have only a single SERINC gene [35]. SERINC5 is highly expressed in multiple tissues in humans including lymphoid tissues but is not induced by interferons [36,37].

SERINC3 and SERINC5 restrict replication of HIV-1 variants lacking the viral accessory protein Nef [36,37]. Analysis of transcriptional profiles or virion proteomes in the presence or absence of Nef established SERINC3 and SERINC5 as targets of Nef, and identified SERINC5 as having more impact on infection by Nef-deficient HIV-1 [36,37]. In the absence of Nef, SERINC5 is incorporated into virions in producer cells [36,37,38]. The Nef block to SERINC5 incorporation likely involves vesicular trafficking, endolysosomal degradation, and a role for polyubiquitination in the targeting of SERINC5 to the endolysosomal vesicular bodies [36,37,39,40,41,42]. The sensitivity of SERINC5 to Nef-mediated antagonism maps to the last of the protein’s cytoplasmic loops [43,44].

SERINC5 is also antagonized by the glycogag (g-gag) of MLVs and S2 of EIAV [36,37,40,45,46,47]. G-gag is a Type II transmembrane protein that is expressed via an alternative start codon upstream of *gag* in some MLVs. Both g-gag and S2-mediated downregulation of SERINC5 levels were shown, like Nef, to function through the endolysosomal system [36,37,40,47]. This convergent evolution of SERINC5 antagonism in different retroviruses is noteworthy in light of the lack of shared sequence or structural homology between Nef, S2, and g-gag.

SERINC5-mediated restriction of HIV-1 is virus Env-type dependent, suggesting that SERINC5 restriction could be at the level of viral entry (Figure 1) [36,37,48,49]. In accordance with this finding, viral reverse transcription in target cells is reduced and viral core delivery to the cytoplasm is blocked in the presence of SERINC5 [36,37,50]. Moreover, fluorescent microscopy of single viral particles as well as super-resolution fluorescent imaging showed that the packaging of SERINC5 into viral particles leads to inhibition of fusion between the virus and the target cell [51,52].

SERINC5 orthologs from multiple mammals as well as amphibians and fish can restrict HIV-1 infection [43,45,53,54,55]. This is in line with the high degree of conservation in the primary amino acid sequence of SERINC5 in vertebrate lineages, and this conservation is reflected in the fact that SERINC5 is under purifying rather than positive selection in primates, suggesting that preservation of its cellular function cannot tolerate alterations [56].

### 2.2. Post Entry

**Figure 3:**
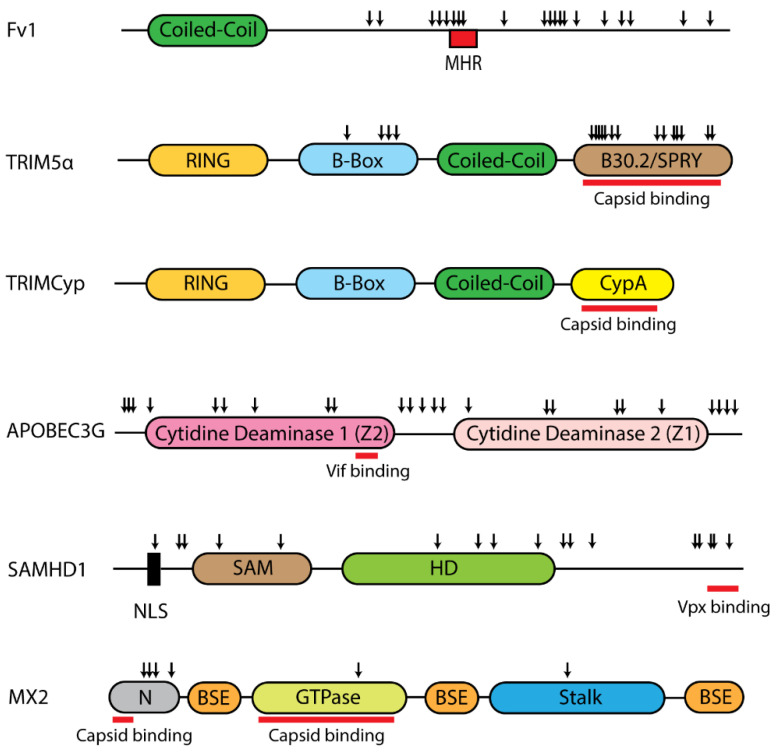
Domain organization of retroviral post-entry restriction factors. Schematic representation identifies positively selected residues (down arrows) found through analyses of Fv1 in rodents and primate genes for TRIM5α, APOBEC3G, SAMHD1 and MX2 [64,65,66,67,68]. The red bars indicate the binding regions for Vif in APOBEC3G, Vpx in SAMHDI and capsid in TRIM5α, TRIMCyp and MX2 [69,70,71,72,73,74]. MHR, major homology region; CypA, cyclophilin A; BSE, bundle signaling element; NLS, nuclear localization signal; N, unstructured amino terminal domain.

After traversing the cell membrane, the retroviral capsid begins to uncoat and reverse transcription (RT) produces a DNA copy that is then transported into the nucleus where it integrates into host chromosomes. Multiple host factors can interfere with these processes at or after RT (Figure 1). These factors differ in domain structure and show signatures of positive selection that align with regions that are either important for restriction or are targeted by viral antagonists (Figure 3).

#### 2.2.1. Fv1

Fv1 was the first retroviral restriction factor to be discovered [57]. Pioneering experiments with different isolates of Friend MLV showed that susceptibility to these isolates varies among inbred mouse strains [57,58]. Specifically, NIH Swiss mice with the *Fv1^n^* allele are permissive to MLVs classed as N-tropic but not to B-tropic MLVs, while BALB/c mice (*Fv1^b^*) are more permissive to B-tropic MLVs [58,59]. Other mouse strains and *Mus* species carry alternative alleles that restrict other MLV variants such as *Fv1^nr^* in 129 mice, *Fv1^d^* in DBA, and other restrictive and nonrestrictive variants in wild mouse species [60,61]. Almost three decades after the discovery of *Fv1* restriction, the responsible gene was identified using positional cloning and shown to have homology to the *gag* gene of an ancient ERV family termed ERV-L [62,63].

The Fv1 block occurs after reverse transcription but before the integration of the viral DNA into the host genome (Figure 1) [75]. Constitutive expression of *Fv1* is low and not IFN-inducible, and it can be saturated by high virus titers [76]. Fv1 restriction targets the capsid protein of the virus, and specific residues involved in restriction have been identified in the target region of the virus capsid as well as in Fv1 [60,61,77,78]. Fv1 interaction with the capsid requires the assembly of a higher-order capsid structure suggesting interference with the virus uncoating process [79].

*Fv1* is found in all but the most basal species in the *Mus* phylogenetic tree, and is missing in the rat genome, so it was initially thought that *Fv1* was acquired shortly after the origins of the *Mus* genus [62,80,81]. Taking advantage of the availability of whole-genome sequenced species, we showed that *Fv1* entered the genome of rodents much earlier than previously thought [64]. These findings indicate that the ERV ancestor of *Fv1* was fixed in the common ancestor of the rodent families *Muridae*, *Cricetidae,* and *Spalacidae* at least 45 million years ago [64,82,83]. Although *Fv1* is lost or substantially mutated in a variety of rodent lineages, the *Fv1* open reading frame (ORF) is present in several branches of the rodent family *Muridae* [64,82].

The accelerated evolution of *Fv1* led to its loss in several lineages but also established signatures of strong positive selection (Figure 3) [64,80,82]. We and others have shown that *Fv1* is evolving under positive selection in *Mus* as well as other lineages in *Muridae* [64,80,82]. Some of the residues evolving under positive selection determine Fv1 restriction suggesting that exposure to retroviral pathogens contributed to this evolution [64,78,80,81,82]. *Fv1* has thus been evolving under positive selection much longer than the exposure of mice to the MLVs that initially defined *Fv1* restriction, and subsequent analyses showed that some *Fv1* variants have demonstrated restriction activity against retroviruses in other genera including foamy viruses and lentiviruses (Table 1) [25,80,81].

#### 2.2.2. TRIM5

In the late 1990s, Fv1-like early blocks to the replication of HIV-1 and MLV were observed in various primate and other mammalian cell lines [84,85]. The responsible factor was identified as TRIM5α [86]. *TRIM5* belongs to the tripartite motif (TRIM) family of genes that encode E3 ubiquitin ligases. The human genome contains more than 80 TRIM family members with roles in various functions ranging from autophagy and innate immunity to cellular differentiation [87,88]. The TRIM genes are named after the three main domains they encode; RING, B-Box, and coiled-coil (Figure 3) [89]. A subset of the TRIM family of proteins, including TRIM5, also contains a fourth domain called SPRY or B30.2 [89]. The restriction factor TRIM5α is encoded by the longest isoform of the primate *TRIM5* gene and includes a SPRY domain [86].

TRIM5 contributes to the species-specific post-entry restriction of retroviruses in several mammalian species [90]. Like Fv1, TRIM5 blocks retroviral replication after entry but before integration (Figure 1) and targets at least one of the same capsid residues as Fv1 [84,86]. Phylogenetic analysis of *TRIM5* orthologs in primates revealed that this gene has been under positive selection [65]. The rapidly evolving residues are concentrated in a “patch” in the SPRY domain and at least one of those sites was subsequently shown to be critical for restriction [65,91]. In fact, TRIM5 binds to the viral capsids of incoming virus through its SPRY domain [69,70]. This leads to a strong block at reverse transcription (Figure 1) [86]. TRIM5 binding to viral capsid occurs only when the capsid molecules are assembled into a higher-ordered structure, and TRIM5 multimers then form a hexagonal net on the viral capsid core [70,92,93,94,95,96,97]. In contrast to its rhesus macaque ortholog, human TRIM5α is a weak restriction factor against HIV-1, but can become a significant contributor to the IFN-mediated inhibition of HIV-1 which likely involves activation of immunoproteasomes [86,98,99,100,101,102].

Like other TRIM proteins, TRIM5 is an E3 ubiquitin ligase owing to the presence of its RING domain [89]. This TRIM5 feature suggested that the ubiquitin-proteasome pathway may be involved in the TRIM5-mediated restriction of retroviruses and in fact, TRIM5 is a short-lived protein that goes through self-ubiquitination [86,103,104]. Hence one proposed mechanism for TRIM5-mediated restriction involves TRIM5 multimers binding to the viral capsid lattice and recruiting proteasomes that degrade the viral core and its components [104,105,106]. Interestingly, both the addition of proteasome inhibitors and the introduction of point mutations in the RING domain of TRIM5 that disrupt self-ubiquitylation restores reverse transcription, but these additions do not restrict virus infection suggesting that TRIM5 may impose subsequent blocks to viral infection [70,104,106,107].

Due to its ability to recognize retroviral capsid structure, TRIM5 was, until recently, thought to be a retrovirus-specific restriction factor. However, TRIM5α of humans and rhesus macaques can inhibit the replication of some flaviviruses by promoting ubiquitination and subsequent degradation of flavivirus protease [102].

TRIM5 from multiple mammalian species has antiviral activity and presents a complex evolutionary history marked by gene duplications, losses, and fusions, some of which have been linked to retroviral resistance [108,109,110,111,112,113,114,115,116,117,118]. The first such example of fusion associated restriction was identified in owl monkeys, where HIV-1 restriction is caused by the LINE-1-mediated retrotransposition of an intronless copy of the cyclophilin A (CypA) gene into the 3′ end of the TRIM5 gene to generate a TRIMCyp fusion protein [108]. This fusion replaces the capsid-binding SPRY domain with the capsid-binding CypA domain (Figure 3) [108]. Independent CypA insertions creating similar but structurally different *TRIMCyp* fusion genes are found in several mammalian lineages, including old world monkeys, tree shrews and rodents, examples of the remarkable convergent evolution of retrotransposition-driven gene fusion in disparate lineages, some of which have demonstrated antiviral activity [112,115,116].

In addition to its function as a retroviral restriction factor, TRIM5 can act as a pattern recognition receptor of the retroviral capsid and activate innate immune signaling pathways [119], although this function may not be uniform in different species [116,120,121]. TRIM5α binding to the viral capsid core initiates a cascade that leads to polyubiquitination of TRIM5α and activation of activator protein 1 (AP-1) and nuclear factor kappa B (Nf-κB) pathways as opposed to the monoubiquitination that happens in the absence of viral infection [122]. This distinct ubiquitination pattern is thought to act in a way that specifically activates the innate immune response only in the presence of virus infection [122].

#### 2.2.3. APOBEC3G

*APOBEC3G* belongs to the apolipoprotein B mRNA editing enzyme catalytic polypeptide-like/activation induced cytidine deaminase (*APOBEC/AID*) family of genes [123]. The 11 members of the *APOBEC/AID* family in humans include *AID*, *APOBEC1*, *APOBEC2*, *APOBEC4*, and seven clustered paralogs of *APOBEC3A-G*. All members of this family except for *APOBEC2* and *APOBEC4* can catalyze the deamination of cytosine to uracil in single-stranded DNA/RNA [123].

APOBEC3G (A3G) was originally identified as a restriction factor for its ability to inhibit the replication of Vif-deficient HIV-1 [124]. Both human A3G and its mammalian orthologs also restrict other lentiviruses and other retroviruses [125,126,127,128,129,130,131,132,133]. A3G is packaged into viral particles and, in subsequently infected cells, it can catalyze the deamination reaction on newly formed viral single-stranded DNA during reverse transcription [134,135]. This leads to the accumulation of G-to-A mutations on proviral DNA which can produce defective viral proteins and non-infectious viral particles [134,135]. A3G can also block reverse transcription independently of its deaminase function [136,137,138]. Moreover, this deaminase-independent block to reverse transcription of Moloney MLV and mouse mammary tumor virus is the major factor in virus restriction by mouse APOBEC3 (mA3) in vivo [139,140,141,142,143]. mA3 can also block the proteolytic processing of MLV *gag* and *gag-pol* and some mA3 proteins incorporate an extra exon that decreases translation efficiency [144,145].

In addition to A3G, several other members of the A3 family can restrict HIV-1 to varying degrees including A3C, A3D, A3F, A3H [125,146,147,148,149,150,151]. Notably, natural polymorphisms of A3C, A3F and A3H in human populations can lead to different restriction profiles against HIV-1 [132,133,147,148,152]. In the case of A3C, a single haplotype found in African populations is the only known variant capable of blocking Vif-deficient HIV-1 [132,148]. Moreover, Both A3F and A3H are highly polymorphic in humans with several variants showing anti-HIV-1 activity [133,146,147,152,153,154,155,156].

Overexpression of APOBEC/AID proteins in transgenic mice can be mutagenic and oncogenic, so expression levels must be regulated [157]. *APOBEC3* (*A3*) genes are expressed at higher levels in hematopoietic cells than the cells of other lineages [158]. Most *A3* genes show high expression levels in T cells, the main target of HIV infection, and the expression of multiple members of the *A3* gene family can be induced by IFNα [158,159]. In addition, mA3 levels are elevated in mice that have an MLV LTR inserted into this gene, another example of exapted ERVs with an antiviral role [160].

A3G was the first restriction factor shown to be counteracted by a retroviral accessory protein. The antiviral activity of the A3 proteins is antagonized by the lentiviral Vif protein [124]. Vif degrades A3G by recruiting an E3 ligase complex that is composed of Cullin5, Elongin B, Elongin C, and Rbx1 [161]. This leads to polyubiquitination and eventual proteasomal degradation of A3G [161]. Vif recruitment and formation of the E3 ligase complex also require the transcription cofactor CBF-β (core-binding factor subunit beta) which stabilizes Vif binding to A3G and the E3 ligase complex [162,163,164,165]. In addition to Vif as an antagonist of human A3G, the MLV g-gag can counter the mA3 restriction of MLV replication [140,166,167]. Unlike the degradative impact of Vif on A3G, g-gag antagonizes mA3 indirectly by stabilizing the viral core and preventing A3 access to the viral DNA/RNA by shielding the viral reverse transcriptase complex [167]. Apart from g-gag, mA3-mediated restriction of MLV replication is also be counteracted by the viral p50 protein, produced by alternative splicing of *gag*, which interacts with mA3 and prevents its packaging into newly produced virions [168,169].

While the *APOBEC/AID* gene family likely originated in early vertebrates, *A3* genes are only found in placental mammals [170,171]. The *A3* paralogs are tightly clustered in a conserved locus, but the copy number of the *A3* genes varies greatly among mammals. For example, while most rodents only have a single *A3* gene, the *A3* locus saw an expansion in primates and a recent study described the acquisition of new *A3* copies in primate genomes through retrotransposition, some of which are active [172,173]. The genomic structure of these *A3* paralogs and orthologs also varies as they can contain one or two zinc-coordinating domains, named according to sequence homology as Z1, Z2, and Z3, but only one Z domain per gene has deaminase activity [174]. In the case of human A3G, the C-terminal cytidine deaminase domain (Z1) has catalytic activity [174].

Phylogenetic and computational analyses revealed that *A3G* has evolved under positive selection in primates and a subsequent analysis of mammalian *A3* genes found signatures of positive selection at several sites concentrated in the Vif binding region in loop 7 of the N-terminal A3Z2 domain involved in substrate recognition [66,71,175]. The single copy of *mA3* is also under positive selection in *Mus,* and the positively selected residues in the catalytically active A3Z2 domain line the substrate groove that accommodates nucleic acids [160]. The demonstration that A3 proteins can also more broadly restrict replication of endogenous retroviruses and retrotransposons, indicate that A3 mutators have been in conflict with retroelements throughout mammalian evolution [175,176,177,178,179]. Furthermore, A3G has been demonstrated to have broader antiviral activity that can block HBV, replication of which includes a reverse transcription step [180,181,182].

#### 2.2.4. SAMHD1

Sterile alpha motif and histidine/aspartic acid (HD) domain containing protein 1 (SAMHD1) is an ISG that functions as a triphosphohydrolase and regulates the levels of intracellular deoxynucleoside triphosphates (dNTPs) [183]. SAMHD1 was first identified as a restriction factor that blocks HIV-1 infection in myeloid and dendritic cells and was later linked to HIV-1 restriction in resting CD4-positive T cells [184,185]. SAMHD1 blocks HIV-1 early in the replication cycle (Figure 1), as its dNTPase activity leads to the depletion of the intracellular dNTP pools available for viral reverse transcription [183,184,186]. When SAMHD1 is depleted from myeloid or dendritic cells, there is an increase in late reverse transcription products, consistent with SAMHD1 inhibition of HIV-1 prior to nuclear import [184,185,186].

The HIV-1 restriction activity of SAMHD1 is limited to non-dividing cells, as these cells have lower levels of intracellular dNTPs than dividing cells [183,184,185]. This aligns with the observation that the phosphorylation status of SAMHD1 at residue T592 is linked to cell cycle regulation [187]. Phosphorylation is accomplished upon S phase entry by the cyclin-dependent kinases (CDKs), CDK1 and CDK2 together with cyclin A2 [188]. Following M phase exit, SAMHD1 is dephosphorylated by the phosphatase PP2A-B55α [187]. The phosphorylation status of SAMHD1 is linked to its anti-retroviral activity, although phosphomimetic mutants of SAMHD1 still decrease dNTP pools but lack anti-lentiviral activity suggesting that there may be a dNTPase-independent restriction function by SAMHD1 [189,190,191,192].

SAMHD1 restriction of lentiviruses is counteracted by two related viral accessory proteins found in different lentiviruses: Vpx in HIV-2 and the primate lentiviruses SIVsm, SIVmac, and SIVrcm, and the Vpr variants in some SIVs [67,184,186,193,194]. Vpx mediates degradation of SAMHD1 through a proteasome-mediated mechanism [74,184,186]. Vpx interacts with the C-terminal domain of SAMHD1 (Figure 3) and recruits the Cullin4-DCAF E3 ubiquitin ligase complex [74,184,186,195]. This leads to polyubiquitination and eventual proteasomal degradation of SAMHD1. Some other lentiviruses, like HIV-1 and its close relative SIVcpz, do not encode Vpx and are thus unable to counteract SAMHD1-mediated restriction [184]. The in vivo consequences of this antagonism of SAMHD1 restriction are unclear as the absence of Vpx in HIV-1 and some SIVs may be beneficial to these viruses by providing a mechanism for immune evasion. This is because unlike HIV-2, HIV-1 cannot efficiently infect dendritic cells and therefore these cells fail to induce IFNβ, so no broad antiviral response can be activated [184,196,197]. However, while SIVmac, which expresses Vpx, can readily infect dendritic cells, it causes a pathogenic infection in macaques suggesting that disease induction involves factors other than immune evasion and Vpx interference [198].

*SAMHD1* has evolved under positive selection in primates as well as other mammals, and some of these positively selected sites overlap with the Vpx interaction sites (Figure 3) [67,193,194,199]. *SAMHD1* orthologs from cats, horses, and cows also show dNTPase activity [200,201]. Both feline and human orthologs inhibit feline immunodeficiency virus (FIV) when overexpressed, and human SAMHD1 also inhibits equine infectious anemia virus (EIAV) and SIV [200,202]. While alpha-, gamma-, and betaretroviruses cannot productively infect non-dividing cells, the addition of exogenous Vpx before infection leads to the increase of late RT products of MLV, Mason Pfizer monkey virus (MPMV) and Rous sarcoma virus (RSV), but not foamy virus, which largely completes reverse transcription prior to target cell entry [202]. In addition to its restriction of lentiviral replication, SAMHD1 inhibits HTLV-1 (human T cell leukemia virus type 1) in non-dividing monocytes [203]. While it remains to be seen whether SAMHD1 has any role in restricting non-lenti retroviruses in vivo, none of the studies performed so far has shown antagonism of SAMHD1 by any of these other retroviruses.

SAMHD1 also restricts HBV, a member of the hepadnavirus family as well as multiple double-stranded DNA viruses, including herpes simplex virus-1 (HSV-1), mouse and human cytomegalovirus, and vaccinia virus [204,205,206,207,208,209]. The dNTPase function of SAMHD1 is required for the restriction of HBV while the inhibition of HSV-1 and vaccinia virus replication was only observed in non-dividing cells [204,205]. Moreover, as shown for retroviruses, phosphorylation at T592 also relieves the inhibitory impact of SAMHD1 on HBV [205,206,210]. This ability to inhibit multiple families of viruses suggests that SAMHD1-mediated depletion of dNTP pools has been adapted to provide a broad innate immune mechanism against viral infection in mammals.

#### 2.2.5. MX2

Myxovirus resistance (MX) proteins are dynamin-like GTPases that are found in most vertebrates [68]. Humans, like most mammals, encode two MX genes: *MX1* and *MX2* [68]. Structural analyses of human MX1 and MX2 proteins reveal a similar domain architecture, despite having only 63% amino acid identity, with a globular GTPase domain connected to the C-terminal stalk domain via a flexible bundle signaling element (Figure 3) [211,212]. Human MX2 was initially described as an HIV-1 restriction factor activated by IFNα [213,214]. MX2 expression has no impact on late reverse transcription products but results in decreased levels of HIV-1 2-LTR circles and integrated proviral DNA [213,214,215]. These findings place MX2-mediated restriction of viral replication after reverse transcription but before integration (Figure 1) [213,214,215]. While the precise mechanism of MX2 inhibition of HIV-1 infection remains to be determined, the viral capsid is a critical target of MX2 restriction, since capsid-specific replacement mutations can escape MX2-mediated inhibition [213,214,215,216,217]. MX2 interacts with in vitro assembled capsid and capsid-nucleocapsid structures and two binding domains have been identified in the N-terminus and in the GTPase region (Figure 3) [72,73,218,219,220].

The determination that MX2 inhibits viral nuclear import is based on the analysis of the two transcriptional isoforms in humans that result from alternative use of an internal start codon [221]. The shorter isoform lacks the N-terminal nuclear localization signal and does not show any antiviral activity, but interferes with the restrictive activity of the longer isoform through competitive capsid binding [72,213,214]. Moreover, the longer isoform localizes to the nuclear periphery while the shorter isoform is cytoplasmic as is MX1 [222]. These findings, together with the demonstration that MX2 inhibition is after reverse transcription, focused attention on nuclear import and led to the findings that several nuclear import proteins interact with MX2 and are involved in MX2 inhibition of HIV-1 [223,224,225].

*MX2* has been under accelerated evolution in primates [68]. Both *MX* genes have a complex evolutionary history in mammals where they have undergone duplications, losses, and gene conversions between the two *MX* genes [68,226,227]. While humans and other primates have *MX1* and *MX2* orthologs, rodents, except for the squirrel clade, have a duplication of *Mx1* and lack an *Mx2* ortholog [68]. This observation together with the fact that human MX2 does not affect MLV replication suggests that MX2 restriction of retroviruses may be specific to lentiviruses [213,214]. Among the species that are infected by known exogenous lentiviruses, horses carry orthologs of both *MX1* and *MX2* and equine MX2 blocks EIAV replication at the same point of the replication cycle as the block to HIV-1 [68,228,229]. In contrast, cats, infectible by FIV, and rabbits that harbor an endogenous lentivirus, only contain a single *MX* gene in their genome, an ortholog of *MX1* [68].

Like MX1, MX2 inhibits a variety of other viruses including herpesviruses, HBV, and flaviviruses as well as LINE-1 retrotransposons, suggesting that this restriction factor evolved to play a significant role in the mammalian defense against viral invasion [201,228,230,231,232,233,234]. Notably, while the GTPase activity of MX2 is dispensable for its restriction of HIV-1, MX2 mutants that are deficient in GTP binding or hydrolysis are unable to block the infection of herpesviruses [231]. The fact that sites under positive selection in primate MX2 do not coincide with the residues that bind HIV-1 capsid structures suggests that the diversifying evolution that shaped this gene was influenced by its interaction with a broader set of pathogens (Figure 3) [68,218].

### 2.3. Post Integration

Restriction factors that operate in late stages of viral replication have been identified in more recent years. While the two highlighted here, ZAP and SLFN11, do not have well-defined mechanisms of action or known viral antagonists, both are under positive selection.

#### 2.3.1. ZAP

Zinc finger antiviral protein (ZAP) is a broad restriction factor that is encoded by the human gene *ZC3HAV1* (zinc finger CCCH-type containing, antiviral 1). Originally discovered as an inhibitor of MLV replication, ZAP is a member of the poly ADP ribose polymerase (PARP) family although both isoforms of ZAP (ZAP-L and ZAP-S) lack the poly ADP ribosylation activity [235,236]. The four ZAP zinc fingers form a binding pocket for the CpG dinucleotides in viral mRNAs which leads to either their degradation or translational repression (Figure 1) [235,237,238,239,240,241,242,243,244]. While the exact mechanism of the ZAP-mediated viral RNA inhibition is not known, it has been shown that ZAP recruits host proteins such as TRIM25 and KHNYN known for their antiviral actions [245,246,247]. ZAP has evolved under positive selection in primates with rapidly evolving residues concentrated at the PARP-like domain [248]. ZAP also restricts a variety of other viruses rich in CpG dinucleotides, including alphaviruses, filoviruses, and HBV as well as retrotransposons (Table 1) [238,249,250,251,252,253,254,255].

#### 2.3.2. Schlafen11

Members of the Schlafen family of genes, which includes Schlafen11 (*SLFN11*), are differentially expressed during thymocyte development [256]. Schlafens are involved in a variety of processes including the regulation of cell cycle, immune cell differentiation, and virus replication [257,258,259]. These genes are found in mammals as well as a few amphibian and fish species [260]. Humans encode six Schlafen genes that are clustered on chromosome 17, and all human Schlafen genes are ISGs [258]. The ten mouse Schlafen genes do not include a *SLFN11* ortholog.

Human SLFN11 inhibits HIV-1 replication at the level of protein synthesis (Figure 1) [226]. Evidence suggests that SLFN11 interacts with tRNAs and blocks the shift of the composition of the tRNA pool induced by HIV-1 [226,261]. The impact of SLFN11 on codon usage bias does not seem to be specific to HIV-1 as SLFN11 can also inhibit translation of EIAV, flaviviruses, and even non-codon optimized non-viral genes [262,263,264]. Moreover, *SLFN11* has evolved under positive selection in primates, but positively selected residues do not seem to be responsible for the restriction level differences observed among primate orthologs suggesting that either an unknown viral antagonist or a non-viral cellular process may be driving this accelerated evolution [263].

### 2.4. Envelope Processing and Packaging

#### 2.4.1. GBP5

Guanylate-binding protein-5 (GBP5) is a member of a family of small GTPases that can be induced by IFNγ [265]. GBP5 was identified as a potential restriction factor of HIV-1 in an evolutionary screen of human genes under positive selection [266]. GBP5 and, to a lesser extent, its paralog GBP2, inhibit HIV-1 replication by interfering with the activity of cellular protease furin which leads to defective envelope processing and incorporation [267,268]. Mutations in the GTPase domain of GBP5 had no impact on its ability to restrict HIV-1 [267]. While there is no evidence of viral antagonism against the restrictive action of GBP5, it has been suggested that mutations in the Vpu initiation codon may confer an advantage to the virus against this inhibition [267,269]. Inhibition of furin cleavage by GBP5 is also responsible for the inhibition of other viruses including MLV, influenza virus and measles virus, and GBP5 is a major contributing factor to the IFNγ-mediated restriction of respiratory syncytial virus demonstrating that this restriction factor has broad antiviral activity [268,270].

#### 2.4.2. MARCH8

Membrane-associated RING-CH 8 (MARCH8) is a member of a RING finger E3 ubiquitin ligase family with 11 members in the human genome [271,272]. MARCH8 blocks HIV-1 Env incorporation into viral particles through surface downregulation that depends on a tyrosine motif found in the cytoplasmic tail of the viral Env [272,273]. The antiviral target of MARCH8 is not limited to the HIV-1 envelope since the Vesicular Stomatitis Virus (VSV) and Ebolavirus glycoproteins were also downregulated from the cell surface in the presence of MARCH8 [272,274]. Two other members of the MARCH family, MARCH1 and MARCH2, can also inhibit HIV-1 and VSV envelope incorporation, and, unlike MARCH8, expression of MARCH1 and MARCH2 can be induced by type I IFNs [275,276].

#### 2.4.3. IFITMs

Interferon-induced transmembrane proteins (IFITMs) are a family of small transmembrane proteins upregulated by interferon during virus infection that are evolutionarily conserved among vertebrates [277]. The most well-studied member of this family, IFITM3, restricts the replication of a variety of viruses including influenza virus, flaviviruses and HIV-1 [278,279,280,281,282,283,284,285,286,287,288]. The mechanism of this restriction is not fully understood as inhibition targets two different stages of the viral life cycle (Figure 1); restriction is observed when IFITM3 is expressed in target cells where it interferes with virus entry as well as in producer cells where it can decrease production of infectious virus. [279,280,289,290,291,292,293]. At the entry level, IFITM3 interferes with fusion between the viral and celluIar membranes and can also reduce the fusogenic activity of syncytin proteins responsible for trophoblast fusion in placentation [278,289,294,295,296]. IFITM3 is embedded in the membranes of endocytic vesicles and after membrane fusion, IFITM3 traffics infecting viruses to lysosomes [294].

When IFITM3 is expressed in virus-producing cells, it is incorporated into retroviral particles which then show substantially decreased ability to infect target cells [280,290,291,292]. IFITM3 interferes with the cleavage of the HIV-1 Env protein gp160, leading to a decrease in the amount of mature Env in viral particles [291,293,297]. This inhibition of envelope packaging was also observed for ecotropic and xenotropic variants of MLV [293]. That IFITM3 interferes with envelope processing/packaging is consistent with the observation that CXCR4-tropic strains of HIV-1 are more sensitive to IFITM3 and IFITM2-mediated restriction than CCR5-tropic strains [298]. While there is no evidence of antagonism of IFITM3 by any of the HIV-1 proteins, g-gag of Moloney MLV can counteract the IFITM3 restrictive function [293].

The number of genes in the *IFITM* cluster varies among vertebrate genomes [299]. For example, while the human *IFITM* cluster in chromosome 11 is comprised of five genes; *IFITM1*, *IFITM2*, *IFITM3*, *IFITM5* and *IFITM10*, the orthologous cluster in the mouse contains six *IFITM* genes [299]. Signatures of positive selection were found among the primate *IFITM1*, *2* and *3* genes located in this cluster, and such sequence variants were also identified in human populations using haplotype analysis [299,300].

#### 2.4.4. Additional Factors

In addition to the restriction factors described above, other proteins have been shown to inhibit retroviral envelope processing and packaging including the mannose receptor and endoplasmic reticulum alpha-mannosidase I (ERManI).

Mannose receptor is a type I transmembrane protein expressed on the surface of macrophages that acts as a pattern recognition receptor with important roles in pathogen internalization by macrophages (reviewed in [301]). This protein inhibits virus egress and also reduces HIV-1 envelope levels [302,303]. While the expression of mannose receptor is not controlled by type I IFNs, HIV-1 accessory proteins Nef and Vpr can counteract the mannose receptor-mediated block to viral release by reducing the surface expression of mannose receptor [303].

ERManI is a member of the glycoside hydrolase family 47 α-mannosidases and plays a critical role in the endoplasmic reticulum-associated protein degradation (ERAD) pathway (reviewed in [304]). ERManI restricts HIV-1 infectivity by initiating the mitochondrial translocator protein (TSPO)-induced ERAD pathway which leads to the degradation of viral Env [305,306]. While the exact mechanism of this restriction is unknown, mutations that abrogate the catalytic activity of ERManI reverse its inhibitory activity on Env suggesting that ERManI may be involved in glycosylation of HIV-1 Env [306]. In addition to its antiviral activity against HIV-1, ERManI is also involved in ERAD-mediated degradation of HA protein of Influenza virus [307].

### 2.5. Assembly and Release

#### BST2/Tetherin

**Figure 4:**
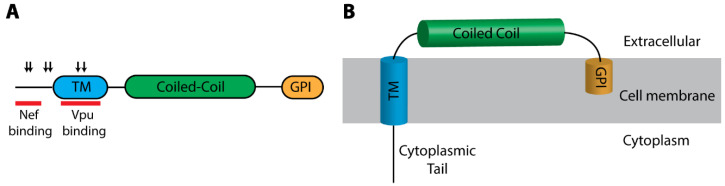
BST2 domain organization and membrane association. (**A**) Schematic diagram of the domain organization of BST2. Positively selected residues are identified by down arrows [345,346]. Regions of HIV-1 Vpu and Nef interaction are indicated with red bars [347,348]. (**B**) Structural representation of BST2 as a transmembrane protein with a GPI anchor. TM, transmembrane domain; GPI, glycosylphosphatidylinositol anchor.

Soon after the discovery of Vpu as an HIV-1 accessory gene, it was demonstrated that HIV-1 without Vpu was not efficiently released from certain cell types [308]. Bone marrow stromal cell antigen 2 (*BST2*) was identified as the responsible ISG by comparing microarray results from various cell types based on their ability to restrict Vpu-deficient HIV-1 [309]. BST2 was originally identified as a surface antigen that is specifically expressed in differentiated B cells [310]. It was later shown that BST2 is expressed in a wide variety of tissues with variable levels of expression [311]. BST2 is a type II single-pass transmembrane protein with a unique domain structure (Figure 4A) [312]. It contains a short N-terminal cytoplasmic tail followed by an alpha-helical transmembrane domain, a coiled-coil ectodomain, and a C-terminal glycosylphosphatidylinositol (GPI) anchor (Figure 4B) [312]. It is the only gene in the human genome that encodes a protein with this structure [313]. This unique topology, with a transmembrane domain at its N-terminus and a GPI anchor at its C-terminus, allows BST2 to act as a bridge between the cell membrane and budding virions, and therefore, BST2 has also been referred to as tetherin [314]. The ability of BST2 to tether budding virions to the cell membrane is not specific to retroviruses, as BST2 blocks release of several other enveloped viruses, including herpesviruses, filoviruses, VSV and SARS coronavirus [315,316,317,318,319,320,321,322,323,324,325].

In addition to cell-free transmission, HIV-1 can also spread between cells via direct contact (reviewed in [326]). While the initial studies on BST2 restriction of retroviruses clearly established a strong block on the cell free transmission of viruses, the role of BST2 on cell-to-cell transmission of HIV-1 is less clear. BST2 can block cell to cell transmission of Vpu deficient HIV-1 from macrophages to CD4 T cells [327], however conflicting results have been reported on BST2-mediated restriction of cell to cell transmission of HIV-1 between T cells [328,329].

The HIV-1 Vpu antagonizes BST2 via multiple mechanisms. Initial studies showed that Vpu interacts with and recruits beta-transducin repeat-containing protein (β-TrCP) to BST2 [330,331,332]. β-TrCP is an F-box protein that makes up the substrate recognition part of the E3 ligase complex with SCF (Skp1-Cullin-F-box) [330,331]. This leads to polyubiquitination and eventual lysosomal degradation of BST2 [330,331]. There is also evidence that Vpu may downregulate BST2 by interfering with its trafficking through the trans-Golgi network [333,334]. In addition to antagonism by the HIV-1 Vpu, the Nef protein of various strains of SIV and the Env of HIV-2 antagonize BST2, and this Nef antagonism of BST2 is important during in vivo infection of rhesus macaques [335,336,337,338,339,340].Unlike their primate counterparts, mouse and rat BST2 orthologs cannot be counteracted by Vpu, Nef or HIV-2 Env [341]. Moreover, mouse BST2 can block spread and pathogenesis of MLV in vivo when induced by type I IFNs or poly (I:C) [342].

Evolutionary studies of *BST2* reveal an ancient origin in early vertebrates [313,343]. BST2 is unusual among retroviral restriction factors in that the amino acid sequence of BST2 shows substantial sequence divergence between different mammalian lineages, although its unique structure is retained [313]. Hence, the ability of BST2 to restrict enveloped viruses relies on its specific domain topology rather than primary amino acid sequence [314]. Within the primate lineage, however, there is a distinctive pattern of sequence divergence. The primate *BST2* evolved under diversifying selection with the positively selected residues concentrated at the cytoplasmic tail and the transmembrane domain, where the Vpu and Nef interacting residues have been mapped (Figure 4A) [344].

## 3. Concluding Remarks

There has been explosive growth in the identification of retroviral restriction factors since the discovery of *Fv1* in 1976, and this set of genes will continue to expand as new screening methods reveal additional cellular proteins that have antiviral activity [99,100,349,350,351]. Most antiretroviral factors are cellular genes that evolved to become part of the innate immune response against viruses, although a subset of restriction factors including *Fv1*, the MLV LTR embedded in mouse *Apobec3*, and the Env-producing *Fv4*, *Rmcf* and *Rmcf2* genes are domesticated ERVs that have no known cellular purpose.

**Figure 5:**
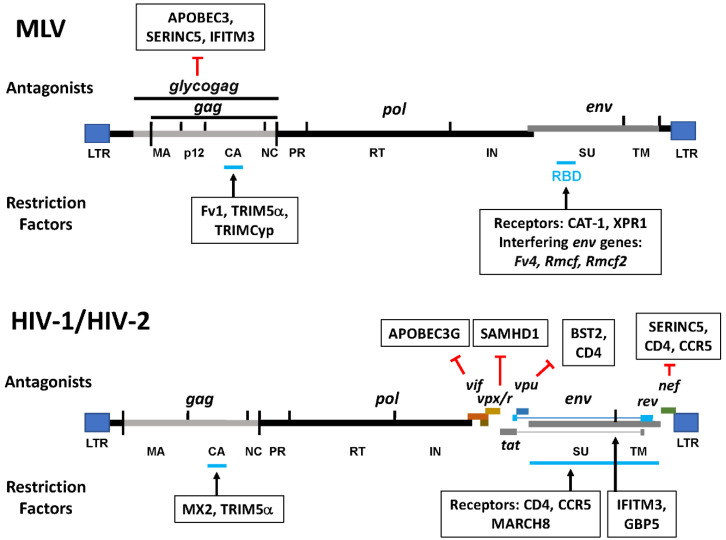
Retroviral targets and virus-encoded antagonists of restriction factors. Viral antagonists and the restriction factors they target are indicated above each schematically represented MLV and HIV-1 genome. Restriction factors and their known viral protein targets are shown below each genome. LTR: long terminal repeat, MA: matrix, CA: capsid, NC: nucleocapsid, PR: protease, RT: reverse transcriptase, IN: integrase, RBD: receptor binding domain, SU: surface protein, TM: transmembrane protein. Vif, Vpu, Vpx/r and Nef are lentiviral accessory proteins.

For some of these factors, restriction operates through interference with cellular processes needed for virus replication, for example, depletion of dNTP pools by SAMHD1 or tRNA pools by SLFN11 [183,257]. Other restriction factors interact with viral nucleic acids such as ZAP which targets CpG islands and APOBEC which induces G to A mutations during reverse transcription [135,247]. A larger subset of restriction factors, however, interact with viral proteins (Figure 5). Their interacting interfaces often display signatures of positive selection and in fact, some of the most intense adaptive evolution in mammalian genes is observed among retroviral restriction factors, marking ratchet-like coevolutionary trajectories of sequential adaptations in restriction factors, their viral targets and viral antagonists [14,352]. These interfaces can either represent viral sites targeted directly by host factors such as capsid residues targeted by Fv1 or TRIM5, or restriction factor sites that interact with viral protein antagonists as illustrated by the inhibition of APOBEC3G by Vif or SAMHD1 by Vpx (Figure 5).

There are many restriction factors that have unexpectedly long historical records of intense diversifying selection, and this argues for broader defensive roles, roles that for many of these factors are now known to extend well beyond their initially defined retroviral targets. Thus, Fv1 was initially defined as a *Mus*-specific factor restricting MLVs but is now known to have inhibitory activity against retroviruses of different genera such as feline foamy virus and some lentiviruses [62,81]. More significantly, many of the restriction factors profiled here like SERINC5, SAMHD1, and BST2 that were originally identified as specifically antiretroviral, show similar inhibitory activity against other viruses including alphaviruses, HBV, and flaviviruses. For at least some of these factors, sites of evolutionary conflict do not necessarily coincide with sites responsible for antiretroviral activity or retroviral antagonist interactions indicating that these adaptations are likely due to challenges by a broader range of viruses. These exceptions include MX2 where positively selected sites are not the ones involved in capsid binding and SFLN11 where positive selection is unconnected to retroviral restriction. This suggests that these rapidly evolving segments result from engagements with multiple viruses.

The rapid evolutionary changes that produce more effective host defenses are not limited to the statistical increase in the rate of non-synonymous mutations, but can also be marked by more dramatic genome reconfigurations including gene duplications and diversifications within specific lineages as seen for TRIM5 which has one copy in human and one to eight copies in rodents, or in APOBEC3 with one gene in mice and seven paralogs in humans, each having different restriction profiles and tissue specific expression patterns [111,116,172]. Genomic insertions can also alter gene expression as shown for mouse Apobec3 and can lead to production of fusion proteins with antiviral activity like TRIMCyp [108,160]. Finally, recurrent gene conversions between related antiviral genes as shown for *MX1* and *MX2* can also contribute to their diversification [68].

While the level of restriction imposed by these factors varies from total blocks to smaller several fold decreases, even marginal inhibitions are amplified during the spreading infections that precede disease. The knowledge gained by studying the interaction between these host restriction factors and their viral targets helps us understand the barriers to cross-species viral transmission, and should also contribute to the development of novel therapeutics that either exploit the inhibitory impact of these restriction factors or target their viral antagonists.

## Figures and Tables

**Table 1:** Properties of Retroviral Restriction Factors.

Restriction Factor	Viral Antagonist *	IFN Induced	Restricted Viruses		Positive Selection *
			Retroviruses	Other *	
CAT1	-	No	Ecotropic MLV	-	No
XPR1	-	No	Nonecotropic MLV	-	Yes
CD4	Vpu, Nef	No	HIV, SIV	-	Yes
CCR5	Vpu, Nef	No	HIV	-	-
Fv4	-	No	Ecotropic MLV	-	-
Rmcf, Rmcf2	-	No	Nonecotropic MLVs	-	-
SERINC5	Nef, Vpu S2 (EIAV) Glycogag (MLV)	No	HIV, SIVs, EIAV, MLV	Alphaviruses, Filoviruses	No
Fv1	-	No	MLV, EIAV, Feline Foamy Virus	-	Yes
TRIM5	-	Yes	Retroviruses	Flaviviruses	Yes
huAPOBEC3G	Vif	Yes	HIV, SIVs, MLV	HBV	Yes
mApobec3	Glycogag, p50 (MLV)		MLV	-	
SAMHD1	Vpx, Vpr	Yes	HIV, SIVs, EIAV, FIV	HBV, Herpesvirus, Vaccinia	Yes
MX2	-	Yes	HIV, SIVs, EIAV, FIV	Herpesvirus, HBV, Flavivirus	Yes
ZAP	-	Yes	HIV, MLV	HBV, Alphaviruses, Filoviruses	Yes
SLFN11	-	Yes	HIV, EIAV, MLV	Flaviviruses	Yes
BST2/Tetherin	Vpu, Nef	Yes	All known retroviruses	Enveloped Viruses	Yes
GBP5	-	Yes	HIV, MLV	Influenza, Zika, Measles	Yes
MARCH8	-	No	HIV	Vesicular Stomatitis Virus, Ebola Virus	-
IFITM3	Glycogag (MLV)	Yes	HIV, MLV	Influenza, Measles	Yes

*-, none or unknown. HIV: Human Immunodeficiency Virus; MLV: Murine Leukemia Virus; SIV: Simian Immunodeficiency Virus; EIAV: Equine Infectious Anemia Virus; FIV: Feline Immunodeficiency Virus; HBV: Hepatitis B Virus.
